# EGFR-targeted therapy results in dramatic early lung tumor regression accompanied by imaging response and immune infiltration in EGFR mutant transgenic mouse models

**DOI:** 10.18632/oncotarget.11021

**Published:** 2016-08-02

**Authors:** Abhilash Venugopalan, Min-Jung Lee, Gang Niu, José Medina-Echeverz, Yusuke Tomita, Martin J. Lizak, Constance M. Cultraro, Robert Mark Simpson, Xiaoyuan Chen, Jane B. Trepel, Udayan Guha

**Affiliations:** ^1^ Thoracic and Gastrointestinal Oncology Branch, National Cancer Institute, Bethesda, MD, USA; ^2^ Developmental Therapeutics Branch, National Cancer Institute, Bethesda, MD, USA; ^3^ Laboratory of Molecular Imaging and Nanomedicine, National Institute of Biomedical Imaging and Bioengineering, Bethesda, MD, USA; ^4^ Mouse Imaging Facility, National Institute of Neurological Disorder and Stroke, National Institutes of Health, Bethesda, MD, USA; ^5^ Laboratory of Cancer Biology and Genetics, National Cancer Institute, Bethesda, MD, USA

**Keywords:** lung cancer, EGFR, TKI, imaging, immune-infiltration

## Abstract

Lung adenocarcinoma patients harboring kinase domain mutations in Epidermal growth factor receptor (EGFR) have significant clinical benefit from EGFR-targeted tyrosine kinase inhibitors (TKIs). Although a majority of patients experience clinical symptomatic benefit immediately, an objective response can only be demonstrated after 6-8 weeks of treatment. Evaluation of patient response by imaging shows that 30-40% of patients do not respond due to intrinsic resistance to these TKIs. We investigated immediate-early effects of EGFR-TKI treatment in mutant EGFR-driven transgenic mouse models by FDG-PET and MRI and correlated the effects on the tumor and the tumor microenvironment. Within 24 hours of erlotinib treatment we saw approximately 65% tumor regression in mice with TKI-sensitive EGFR^L858R^ lung adenocarcinoma. However, mice with EGFR^L858R/T790M^-driven tumors did not respond to either erlotinib or afatinib monotherapy, but did show a significant tumor response to afatinib-cetuximab combination treatment. The imaging responses correlated with the inhibition of downstream EGFR signaling, increased apoptosis, and decreased proliferation in the tumor tissues. In EGFR^L858R^-driven tumors, we saw a significant increase in CD45^+^ leukocytes, NK cells, dendritic cells, macrophages and lymphocytes, particularly CD8^+^ T cells. In response to erlotinib, these dendritic cells and macrophages had significantly higher MHC class II expression, indicating increased antigen-presenting capabilities. Together, results of our study provide novel insight into the immediate-early therapeutic response to EGFR TKIs *in vivo*.

## INTRODUCTION

Lung cancer is the leading cause of cancer related mortality, with an estimated 1.59 million deaths worldwide each year [1]. Somatic mutations were identified in a subset of non-small cell lung cancer (NSCLC) patients who responded to epidermal growth factor receptor (EGFR) tyrosine kinase inhibitors (TKIs), such as erlotinib and gefitinib [2–4]. The most common of these sensitizing mutations are: 1) in-frame deletions in exon 19 that delete the amino acids, leucine, arginine, glutamic acid and alanine (LREA, EGFR^Del^) and 2) a missense mutation that results in the substitution of arginine for leucine at position 858 (EGFR^L858R^). EGFR^L858R^ and EGFR^Del^ mutations activate EGFR constitutively resulting in autophosphorylation of the tyrosine residues in the carboxyl tail of EGFR. The phosphorylated residues serve as docking sites for the recruitment of adaptor proteins. This overall assembly leads to the transmission of signals to downstream pathways, including -MEK-ERK (MAPK) and PI3K-AKT kinase cascades. Together, these lead to pro-survival and anti-apoptotic signals [5, 6]. *In vitro* Hence, the tumors presenting these mutations are addicted to the activation of EGFR. *EGFR*

The first generation EGFR-TKIs demonstrated significant clinical response in patients with tumors driven by EGFR-TKI sensitizing mutations [8–10]. However, patients eventually develop acquired resistance. The most common of these acquired resistance mechanisms is the acquisition of a T790M mutation in the EGFR kinase domain. This mutation increases the ATP affinity of mutant EGFR and causes steric interference with the binding of first generation TKIs [4, 11, 12]. Afatinib is a second generation irreversible ERBB family inhibitor that blocks signaling from ERBB family members, including EGFR and HER2 [13, 14]. Afatinib was shown to target EGFR mutations, including T790M, efficaciously *in vitro* and *in vivo* [13]. However, in clinical trials, afatinib failed to increase overall survival, in patients with acquired resistance to first-generation TKIs [15].

Aberrant EGFR signaling is also inhibited by antibodies which bind to the extracellular domain of EGFR. Cetuximab is a human-murine chimeric anti-EGFR antibody that binds to the EGFR extracellular domain and competitively inhibits ligand binding [16]. Pre-clinical studies using EGFR^L858R/T790M^-driven tumor models have shown that afatinib-cetuximab combination treatment can achieve significant tumor regression [17], a finding further confirmed in patients with acquired resistance to EGFR TKIs [18]. In lieu of the fact that approximately 30-40% of patients harboring TKI-sensitizing mutations still demonstrate intrinsic resistance to EGFR TKIs [19], it is imperative that efforts are directed to finding early treatment biomarkers, as well as, interrogating the early effects of TKI treatment in the tumor compartment and its microenvironment.

The difficulty is that there tends to be a clinically significant delay in adequate monitoring of the therapy's specific physiological effects. In certain pre-clinical models, tumor response has been measured by magnetic resonance imaging (MRI) one week after initiation of treatment [13, 17, 20]. In a number of clinical studies, tumor response was assessed from 4 to 6 weeks after treatment initiation [9, 15, 18]. Studies have also reported rapid symptomatic improvements after one week of treatment initiation [21]. Importantly, patients often reported that they feel better immediately after the initiation of EGFR TKIs. However, these early observations cannot be confirmed in patients, as the follow-up scans are performed 6-8 weeks after initiation of treatment. For a disease as serious as lung cancer, it is important to identify and analyze the immediate treatment responses in pre-clinical models, as these findings may reflect major physiological and biochemical developments that may portend eventual treatment response. These immediate-early treatment responses of tumors may be measured by ^18^F-fluoro-2-deoxy-glucose positron emission tomography (FDG-PET), a very sensitive and clinically relevant approach [22, 23].

Addressing such problems requires close attention to the tumor microenvironment as a whole. Several studies have shown that tumor microenvironment influences anti-tumor treatment responses significantly [24]. Various investigations have demonstrated that chemotherapy in combination with blockade of pathways mediating macrophage recruitment significantly decreases primary tumor development and metastasis [25]. In certain cancers, a lymphocyte increase consecutive to chemotherapy has proven to be an independent predictive biomarker for reduced relapse rate and improved survival [26, 27]. In BRAF^V600E^-driven melanomas, targeted therapies have resulted in increased CD8^+^ T-cell infiltration and anti-tumor responses [28]. Thus, a considerable body of research suggests that analysis of the tumor microenvironment is crucial for understanding treatment response as well as for identifying potential targetable molecular pathways.

In this study, we sought to investigate immediate-early effects of EGFR TKI treatment on tumor regression and tumor microenvironment in mutant EGFR-driven “pre-clinical” genetically engineered mouse (GEM) models. In these models, lung adenocarcinoma is generated by doxycycline-induced lung epithelial expression of transgenic *EGFR*^L858R^, a TKI-sensitive mutation, and by *EGFR*^L858R/T790M^, which accounts for approximately 60% of acquired resistance to EGFR TKIs [29]. We have measured EGFR-TKI responses by FDG-PET, and by MRI, 24 hours after treatment initiation. In *EGFR*^L858R^ mice, MRI demonstrated greater than 65% tumor regression. Likewise, FDG-PET imaging showed a similar immediate-early treatment response. Furthermore, these observations were accompanied by changes in signaling and in the tumor immune microenvironment. Such immediate-early responses, however, were not seen in EGFR^L858R/T790M^-driven TKI-resistant tumors treated with erlotinib or afatinib. A combination of afatinib with cetuximab, however, had a definite intermediary effect. We also investigated the immediate changes to EGFR signaling and the tumor immune microenvironment. We demonstrate a significant increase in specific subsets of immune infiltrates at 24 hours in erlotinib-treated mice with EGFR^L858R^-driven tumors and characterize the immune subpopulations in the tumor microenvironment.

## RESULTS

### Early imaging response to erlotinib treatment in EGFR^L858R^-driven lung adenocarcinoma mouse models

**Figure 1:**
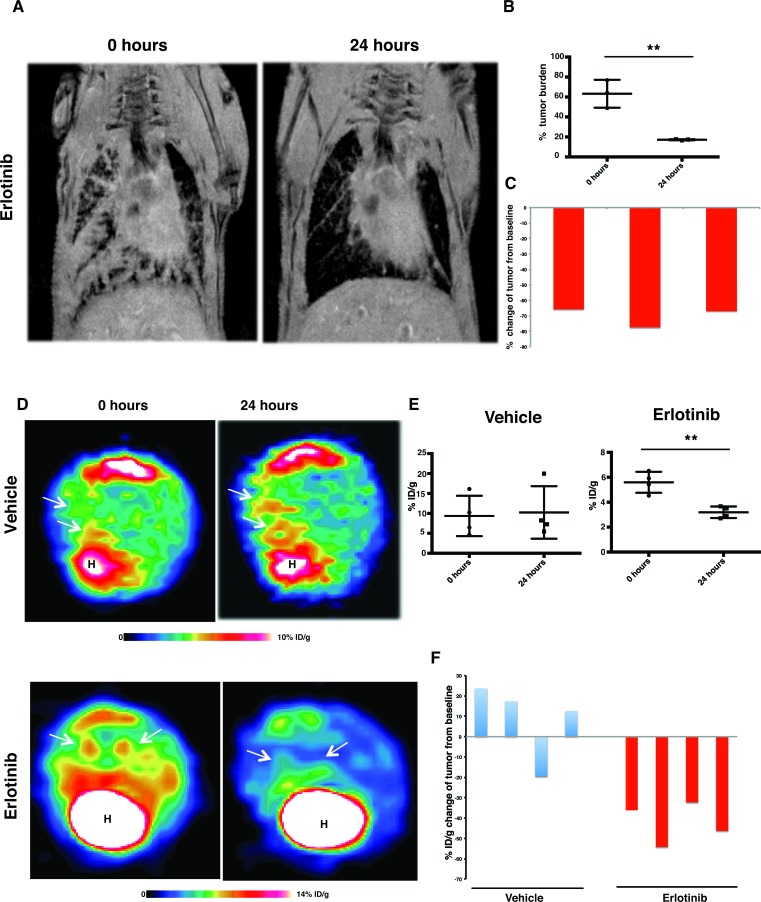
MRI and FDG-PET show a dramatic treatment response within 24hours *CC10*^rtTA^*/EGFR*^L858R^ transgenic mice were fed doxycycline to induce *EGFR*^L858R^-driven lung tumors. **A.**, representative lung MRI of *CC10*^rtTA^*/EGFR*^L858R^ mice at baseline and within 24 hours after the treatment with erlotinib. **B.**, quantitation of tumor burden of mice as measured by MRI. Statistical evaluation was performed by Student's *t* test (**p < 0.01), *n* = 3 and data presented as mean +/− SEM. **C.**, waterfall plot showing tumor response within 24 hours after treatment with erlotinib in EGFR^L858R^-driven tumors, with data shown relative to the baseline scan performed before treatment. **D.**, representative FDG-PET image from *CC10*^rtTA^*/EGFR*^L858R^ transgenic mice at baseline and within 24 hours after treatment with either vehicle or erlotinib. For each animal, the baseline and post-treatment FDG-PET images are depicted on identical scales. White arrows point to lesions. **E.**, plot showing tumor response in EGFR^L858R^-driven tumor tissue to either vehicle or erlotinib treatment, data presented as mean +/− SEM. Measuring the magnitude of response from the baseline treatment and the corresponding p value obtained by Student's *t* test (**p < 0.01), where *n* = 4 for vehicle- and erlotinib-treated cohorts. **F.**, waterfall plot displays tumor response within 24 hours after treatment with either vehicle or erlotinib. (Taken from the baseline scan performed before the treatment.)

Mutant EGFR-induced lung adenocarcinoma mouse models have seen wide use as pre-clinical models for investigation of treatment strategies [4, 20]. However, the early responses to EGFR tyrosine kinase inhibitors are poorly understood. We used doxycycline inducible bi-transgenic (*tetO-EGFR*^mut^ and *CCSP*^rtTA^) lung adenocarcinoma mouse models to study the tumors’ immediate-early response to EGFR TKIs. We treated one cohort of littermates of EGFR^L858R^-driven tumor-harboring mice with 25mg/kg/day of erlotinib and another cohort of littermates with vehicle. The mice were imaged by MRI on day 1 after the first dose of erlotinib and received a second dose three hours prior to the imaging. Those mice treated with erlotinib exhibited dramatic tumor regression (Figure 1A and 1B). Upon erlotinib treatment, tumor burden was reduced by 65% from baseline (Figure 1C). To confirm our results, FDG-PET imaging was used as an early-treatment response marker suitable for clinical use.

We treated another cohort of *EGFR*^L858R^-harboring mice with 25mg/kg/day erlotinib or with vehicle, and subjected these animals to FDG-PET imaging within 24 hours after the first treatment dose. A second dose was administered three hours prior to the imaging. We euthanized these mice immediately after the imaging, for further characterization. As shown by FDG-PET imaging, reduced FDG metabolism in those treated with erlotinib correlated with significant response to erlotinib treatment within 24 hours as shown by the MRI (Figure 1D and 1E). All four erlotinib-treated mice displayed a decreased FDG uptake in tumors. These tumors showed a 32-54% decreased uptake from baseline, but most of the vehicle-treated mice presented no FDG response in tumors as measured by FDG-PET imaging (Figure 1F). These data with erlotinib treatment indicate that, in mutant EGFR transgenic mouse models, EGFR^L858R^ oncogene-addicted tumors dramatically respond as early as 24 hours after EGFR-TKI treatment.

### Early imaging response to afatinib-cetuximab combination treatment in *EGFR*^L858R/T790M^-induced tumors

**Figure 2:**
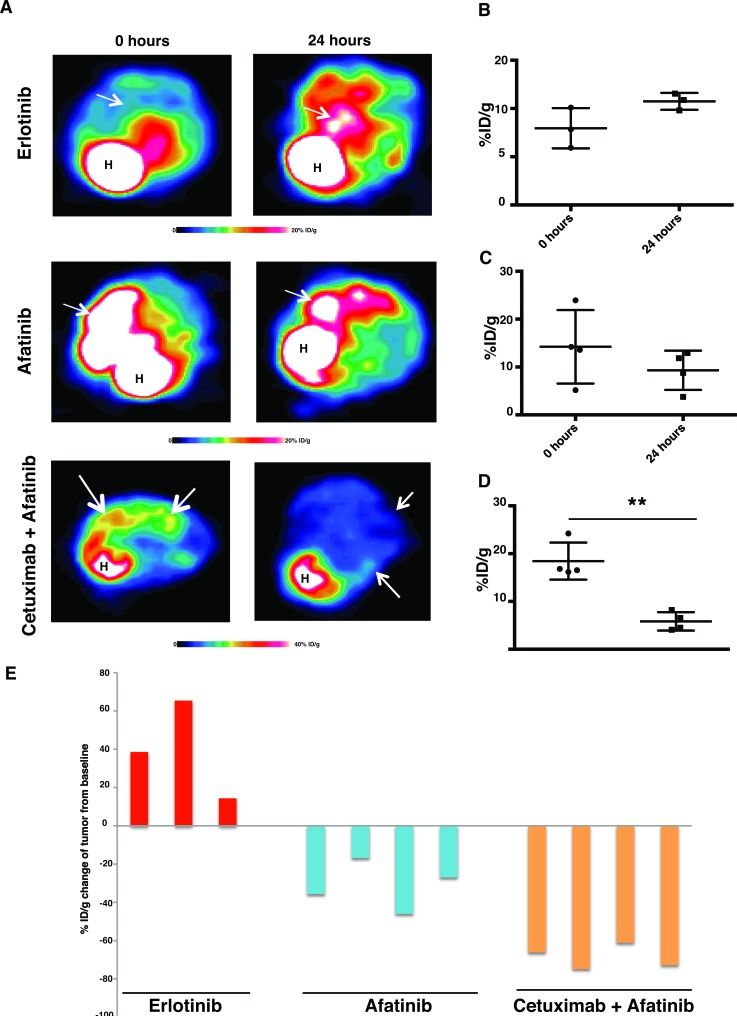
FDG-PET shows a significant early response to afatinib-cetuximab combination treatment *CC10*^rtTA^*/EGFR*^L858R/T790M^ transgenic mice were fed with doxycycline to induce EGFR^L858R/T790M^-driven lung tumors. **A.**, representative FDG-PET image from *CC10*^rtTA^*/EGFR*^L858R/T790M^ mice at baseline and within 24 hours after treatment with either erlotinib monotherapy (*n* = 3), afatinib monotherapy (*n* = 4) or a combination of afatinib and cetuximab (*n* = 4). For each animal, the baseline and post-treatment FDG-PET images are depicted on identical scales. White arrows point to lesions. Tumor responses to erlotinib **B.**, afatinib **C.** and afatinib-cetuximab combination **D.** were plotted and analyzed by Student's *t* test (**p < 0.01) and presented as mean +/− SEM. **E.**, waterfall plot displaying tumor response within 24 hours after treatment, from the baseline scan performed before treatment.

Patients who respond to erlotinib eventually develop resistance by acquiring a T790M gatekeeper mutation in the EGFR kinase domain. Afatinib, a second-generation TKI which can irreversibly bind EGFR, has failed to demonstrate significant clinical benefits in patients harboring the EGFR^T790M^ mutation [30]. However, pre-clinical studies and clinical trials have shown that tumors driven by EGFR^L858R/T790M^ respond to a combination of afatinib and cetuximab therapy [17, 18]. We used FDG-PET imaging, to evaluate the early response of EGFR^L858R/T790M^-driven tumors to erlotinib, afatinib and an afatinib-cetuximab combination (Supplementary Table 1). Combination therapy resulted in the most significant response (Figure 2A). The tumors did not respond to erlotinib alone, rather they showed an increased FDG-PET activity that ranged from 14% to 39%, as compared to the baseline scan (Figure 2B). Likewise, the *EGFR*^L858R/T790M^-transgenic mice treated with afatinib alone displayed only a non-significant trend towards decreased FDG uptake, the range being 16%-35% compared to the baseline scans (Figure 2C). Upon treatment with afatinib-cetuximab combination therapy, however, EGFR^L858R/T790M^-induced tumors exhibited significantly reduced FDG uptake, ranging from 61% to 75% (Figure 2A, 2D and 2E).

### 24-hour treatment response to erlotinib was accompanied by increased cell death and reduced proliferation in EGFR^L858R^-driven lung tumors

**Figure 3:**
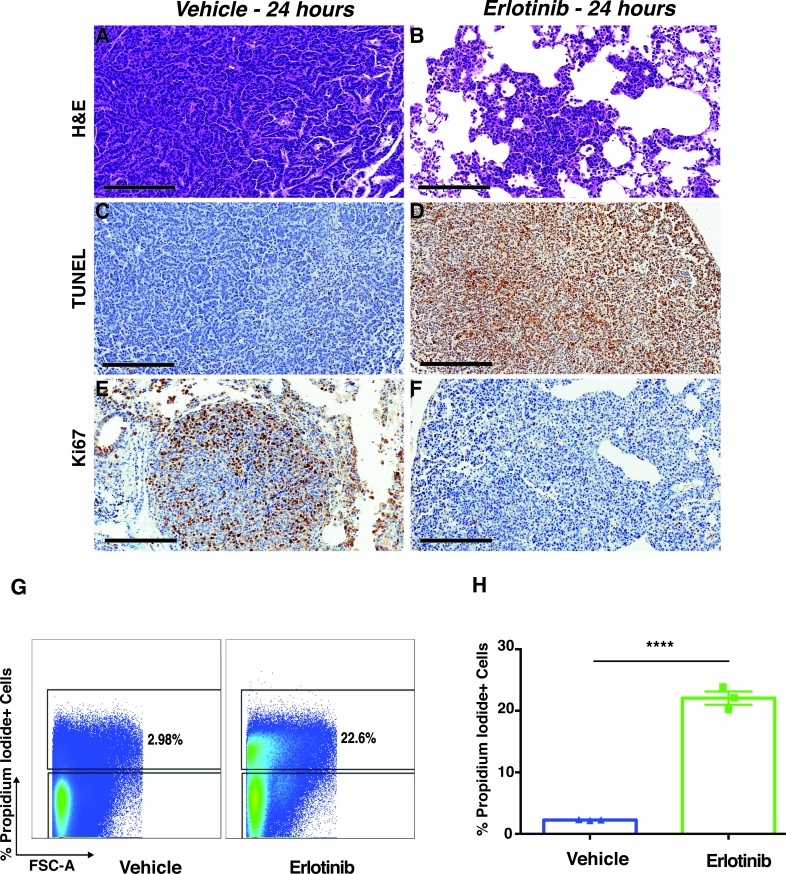
Erlotinib treatment-induced apoptosis in EGFR^L858R^ tumor-bearing mice within 24 hours after treatment Immunohistochemistry of lung-tissue sections from *EGFR*^L858R^ transgenic mice treated with either vehicle (*n* = 4) or erlotinib (*n* = 4). Hematoxylin-and-eosin staining **A.**-**B.**, scale bars, 300 μm), TUNEL staining **C.**-**D.**, scale bars, 200 μm) and Ki67 **E.**-**F.**, scale bars, 200 μm) staining show tumors’ early response to erlotinib treatment. **G.**, Flow-cytometric analysis of EGFR^L858R^-driven tumor tissue after treatment, with either vehicle or erlotinib, within 24 hours after treatment. Live/dead analysis of cells in erlotinib-treated tumors compared to those treated with vehicle. **H.**, Percentage of dead cells (Propidium Iodide^+^) 24 hours after treatment show significantly increased dead cells in erlotinib treated tumors compared to vehicle treated tumors. Data are expressed as a mean +/− SEM. Statistical analysis: ****p < 0.0001 (Student's *t* test), *n* = 3 per treatment group.

To better characterize the dramatic early response to erlotinib seen in the EGFR^L858R^-driven lung adenocarcinoma, we performed histopathological examination on tumor tissues and measured apoptosis, and cellular proliferation, within 24 hours of treatment. Hematoxylin-and-eosin (H&E) staining revealed significant tumor regression in erlotinib-treated mice, compared to vehicle-treated mice (Figure 3A-3B). In addition, there was increased leukocyte infiltration in erlotinib-treated mice (Supplementary Figure 1).

EGFR TKIs elicit cell death through apoptosis [31]. We measured apoptosis in EGFR^L858R^-driven tumor-bearing mice that had been treated with erlotinib. Terminal deoxynucleotidyl transferase-mediated dUTP nick-end labeling (TUNEL) assay results confirmed cell death due to apoptosis in the erlotinib-treated tumor tissues (Figure 3C-3D). Additionally, we used Ki67 staining to assess tumor-cell proliferation occurring within 24 hours of treatment. Proliferation decreased markedly in the erlotinib-treated tumors, whereas the vehicle-treated tumors were unaffected (Figure 3E-3F). In agreement with the TUNEL assay, results of flow-cytometry dead-cell discrimination staining showed a significant increase in dead-cell numbers in EGFR^L858R^-driven tumor-bearing mice treated with erlotinib, (Figure 3G-3H). Altogether, our *in vivo* data show that targeted therapy using erlotinib significantly induced tumor-cell death and decreased proliferation, in EGFR^L858R^-driven lung adenocarcinomas within 24 hours.

### 24-hour treatment response to afatinib-cetuximab combination, but not afatinib or erlotinib monotherapy was accompanied by increased cell death and reduced proliferation in EGFR^L858R/T790M^-driven lung tumors

**Figure 4:**
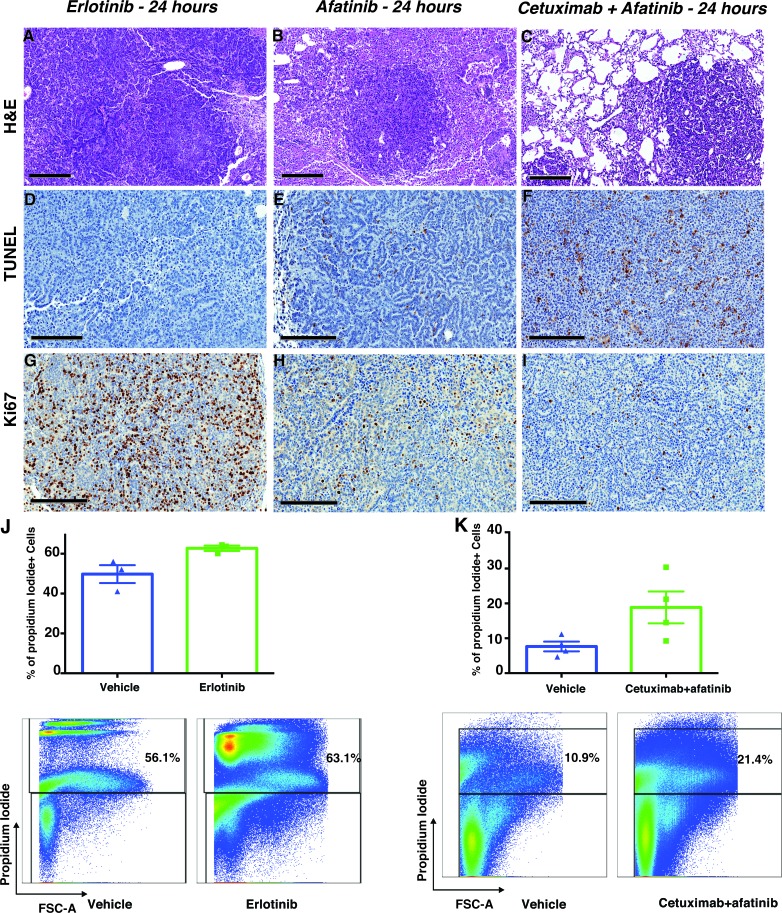
Combination therapy both induced apoptosis and decreased the proliferation of EGFR^L858R/T790M^-driven lung adenocarcinoma The immunohistochemistry of lung adenocarcinoma driven by *EGFR*^L858R/T790M^ transgenic mice treated with either erlotinib (*n* = 3), afatinib (*n* = 4) or an afatinib-cetuximab combination (*n* = 4). The immunohistochemical analysis of hematoxylin-and-eosin staining **A.**-**C.**, scale bars, 300 μm), TUNEL **D.**-**F.** scale bars, 200 μm) and Ki67 staining **G.**-**I.** scale bars, 200 μm) show tumors’ early response to afatinib-cetuximab combination therapy. **J.**, flow-cytometry analysis of live/dead cells was performed from lungs that were harvested within 24 hours after treatment with either vehicle (*n* = 3) or erlotinib (*n* = 3). **K.**, flow-cytometry analysis of live/dead cells was performed from lungs that were harvested within 24 hours after treatment with either vehicle (*n* = 3) or afatinib-cetuximab combination therapy (*n* = 3).

We compared the therapeutic efficacies of three different treatment regimens on EGFR^L858R/T790M^-driven tumors; erlotinib or afatinib monotherapy, and afatinib-cetuximab combination therapy. Here, we primarily focused on histopathological changes. The combination treatment presented a notable reduction in tumor burden, whereas the erlotinib- and afatinib-treated cohorts did not (Figure 4A-4C). TUNEL staining of the combination treated tumors showed a definite increase in apoptosis, whereas apoptosis in the afatinib-monotreated tumors was increased to a lesser extent and there was no apoptosis in the erlotinib single therapy cohort (Figure 4D-4F). Ki67 staining of the combination treated cohort showed a decrease in proliferation and the afatinib monotherapy decreased proliferation but to a lesser extent. (Figure 4G-4I). As compared to the other two treatment regimens, erlotinib-treated tumors did not undergo apoptosis, but exhibited increased proliferation. Consistent with the TUNEL data, flow-cytometry analysis by dead-cell discrimination staining showed more dead cells in tumors treated with combination therapy as compared to erlotinib treatment (Figure 4J-4K), although not statistically significant. Together, these results are consistent with our early imaging observation that only the afatinib-cetuximab combination therapy gave a significant response in EGFR^L858R/T790^-driven tumors.

### EGFR pathway modulation at 24 hours of TKI treatment in EGFR^L858R^ and EGFR^L858R/T790M^ mutant tumors

**Figure 5:**
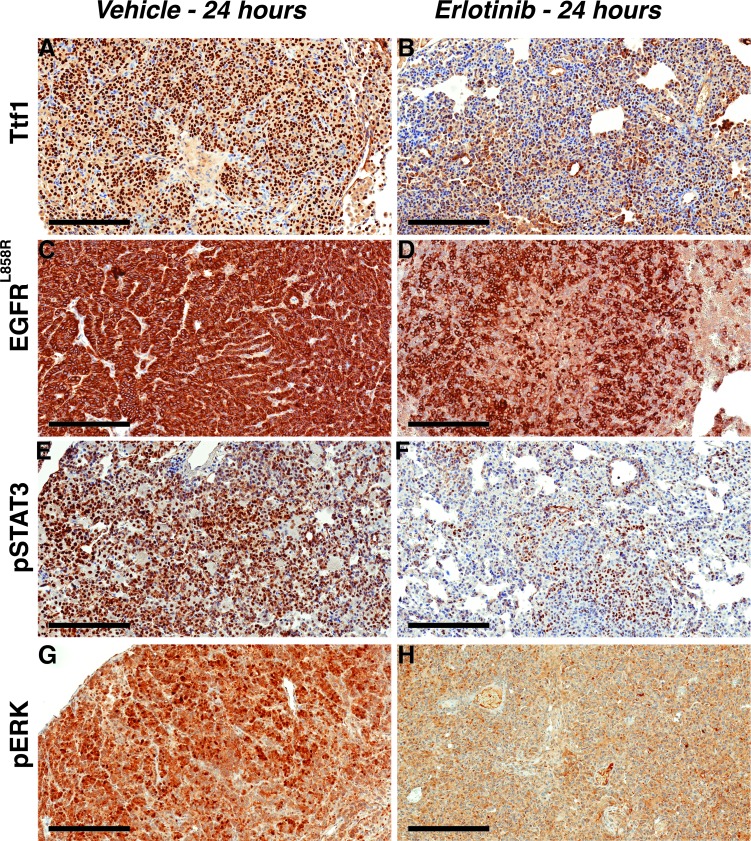
Erlotinib treatment triggers rapid response by decreasing survival signals in EGFR^L858R^-driven lung adenocarcinoma Staining with Ttf1 **A.**-**B.**, EGFR^L858R^ **C.**-**D.** pSTAT3 **E.**-**F.** and pERK **G.**-**H.** shows treatment response to erlotinib. Scale bars, 200 μm.

To gain insight into pathway changes downstream of EGFR in response to early erlotinib treatment, we performed immunohistochemical analyses. Thyroid transcription factor 1 (Ttf1) was used as a marker for type II epithelial cells, which are precursors for lung adenocarcinoma [32]. To assay TKI-induced anti-tumor responses we used EGFR^L858R^ and the EGFR downstream pathway proteins, ERK1/2 (phospho-ERK1/2 Thr 202/Tyr 204), AKT (phospho-AKT Thr 308) and a signal transducer and activator of transcription 3 (phospho-STAT3 Tyr 705) [6, 20]. There was reduced Ttf1 staining in erlotinib treated EGFR^L858R^-driven tumors suggesting a decrease in tumor cells originating from type II epithelial cells (Figure 5A-5B). Likewise, EGFR^L858R^ staining also decreased significantly, confirming that the transgenic mutant EGFR-expressing tumor cells were depleted within 24 hours of erlotinib treatment (Figure 5C-5D). Moreover, there was reduced pERK and pSTAT3 staining indicative of reduced downstream EGFR signaling.

**Figure 6:**
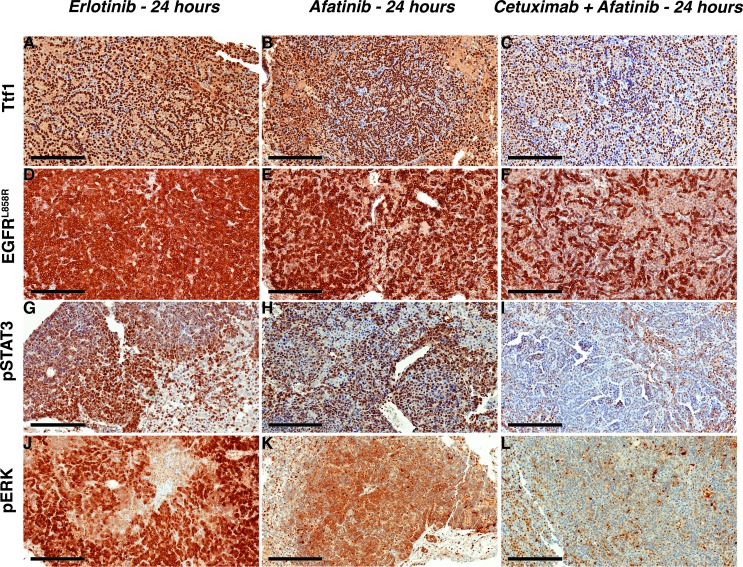
Afatinib-cetuximab combination therapy triggers rapid response by decreasing survival signals in EGFR^**L858R/T790M**^- driven lung adenocarcinoma Staining with Ttf1 **A.**-**C.**, EGFR^L858R^ **D.**-**F.** pSTAT3 **G.**-**I.** and pERK **J.**-**L.** shows treatment response to afatinib -cetuximab combination. Scale bars, 200 μm.

**Figure 7:**
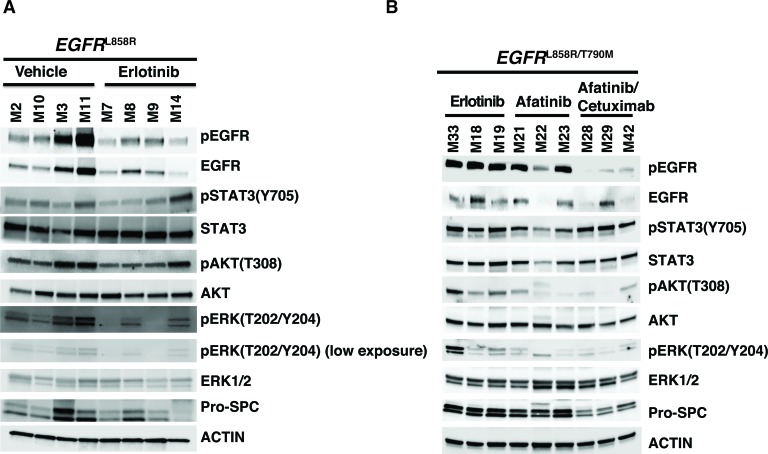
EGFR downstream signaling decreased upon both erlotinib treatment of EGFR^**L858R**^ driven lung adenocarcinoma and afatinib-cetuximab combination therapy treatment of EGFR^**L858R/T790M**^ driven lung adenocarcinoma **A.**, protein lysates derived from EGFR^L858R^ driven tumors, within 24 hours after the treatment with either vehicle or erlotinib were immunoblotted. Membranes were probed using both phospho-specific and total antibodies against EGFR pathway components. Expressions of pro-surfactant C (Pro-SPC) and Actin (loading control) were also examined. **B.**, protein lysates from EGFR^L858R/T790M^ driven tumors after treatment with erlotinib, afatinib or a combination of afatinib-cetuximab were immunoblotted. Membranes were probed using both phospho-specific and total antibodies against EGFR pathway components; Pro-SPC and Actin (loading control).

In EGFR^L858R/T790M^-driven tumors treated with afatinib-cetuximab combination therapy, we observed a marked decrease in Ttf1 and EGFR^L858R^ staining. In contrast, there was no change in either monotherapy. This finding confirmed the tumor-burden reduction advantage of combination *versus* monotherapy (Figure 6A-6F). Likewise, the decrease in pERK and pSTAT3 staining in tumors from mice receiving combination therapy demonstrated reduced EGFR signaling (Figure 6G-6L). To continue our analysis of EGFR signaling, we used Western immunoblot assays. In the EGFR^L858R^-driven tumors, within 24 hours after erlotinib treatment we found reduced EGFR, pEGFR, pAKT, pERK and pSTAT3 expression. (Figure 7A). In the EGFR^L858R/T790M^-driven tumors, within the same time frame, combination therapy resulted in reduced expression of pEGFR, pAKT and pERK (Figure 7B). Thus, both the *EGFR^L858R^* mice treated with erlotinib and the *EGFR^L858R/T790M^* mice treated with afatinib-cetuximab, the dramatic tumor response was accompanied by reduced downstream EGFR signaling.

### EGFR^L858R^-driven tumors displayed increased immune infiltration in response to erlotinib treatment

**Figure 8:**
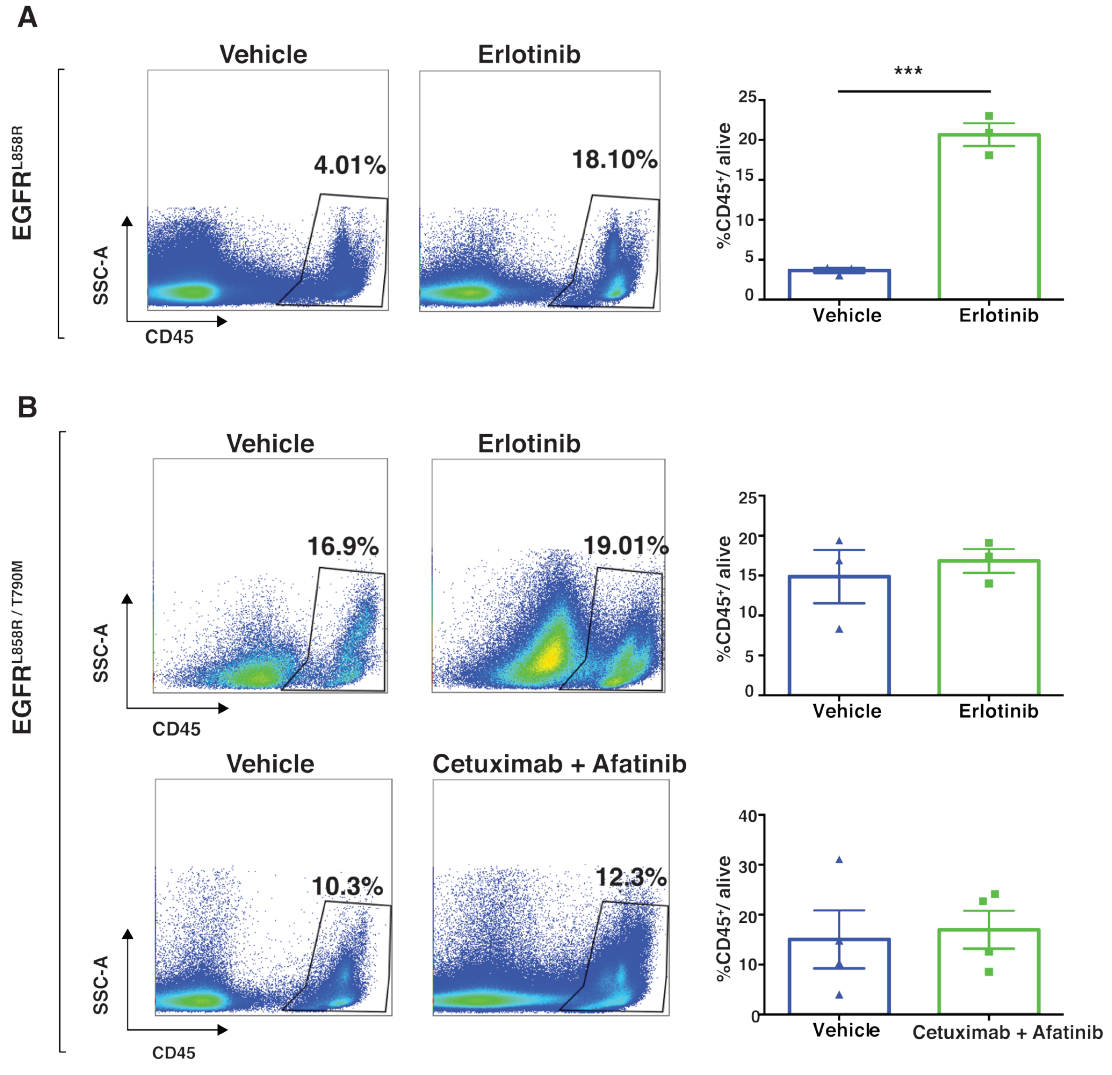
Immune profiling demonstrated increased leukocyte infiltration in EGFR^**L858R**^-driven tumors after treatment with erlotinib **A.**, Flow-cytometry analysis of single cells isolated from vehicle (*n* = 3) and erlotinib-treated EGFR^L858R^-mice (*n* = 3) within 24 hours of treatment. Numerical data indicate the percentage of CD45^+^ leucocytes among the total live cells analyzed. Representative plots from each group are shown and data are represented as mean +/− SEM. Statistical analysis: ***p < 0.001 (Student's *t* test). **B.**, Flow-cytometry analysis of tumor cells from *EGFR*^L858R/T790M^ transgenic mice that were treated with either vehicle, erlotinib or an afatinib-cetuximab combination.

**Figure 9:**
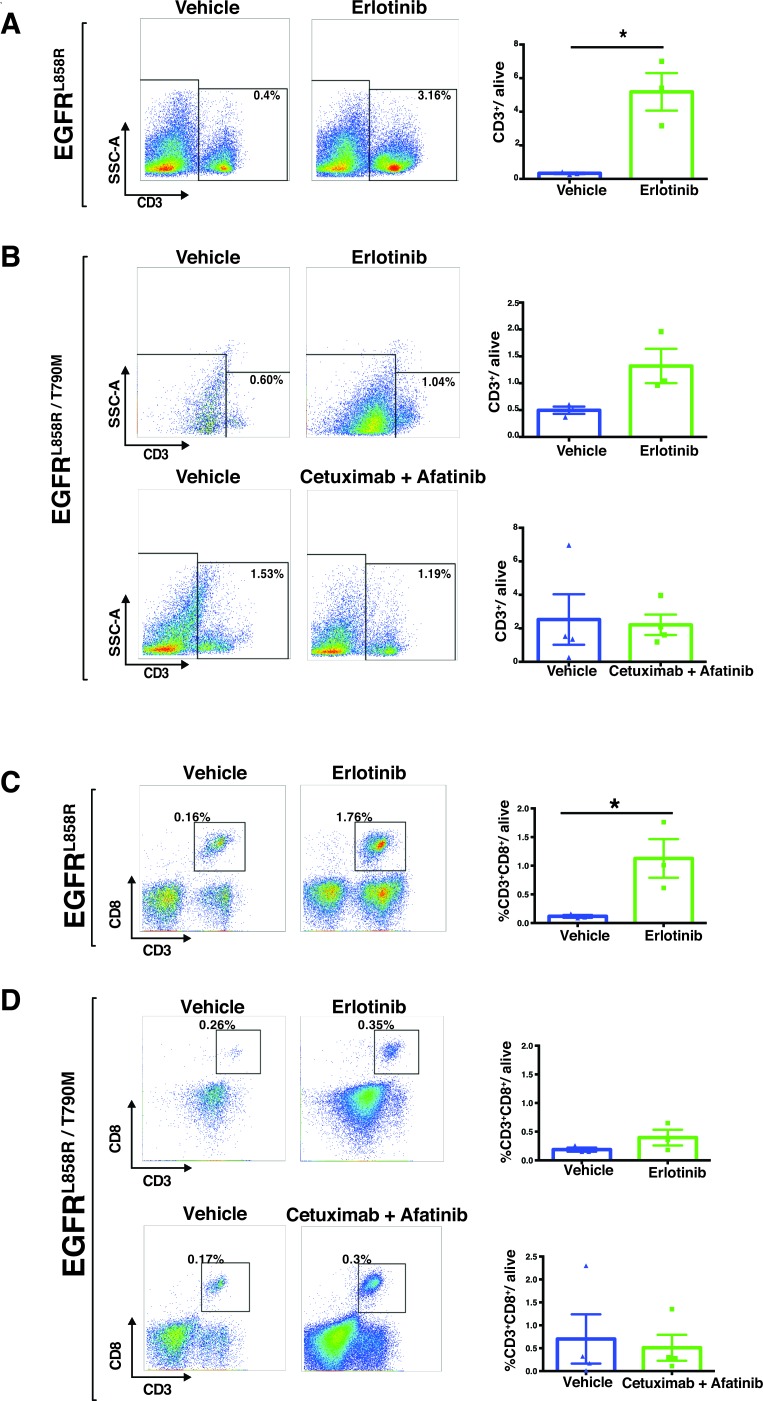
Increased T lymphocytes in EGFR^**L858R**^-driven tumors treated with erlotinib Lung tumors from *EGFR*^L858R^ and *EGFR*^L858R/T790M^ transgenic mice were excised within 24 hours after treatment as indicated in the figure and subjected to flow cytometry **A.**-**D.** Percentages of cells were shown relative to total live cells. **A**, T cells (CD45^+^ CD3^+^), specifically CD8^+^ T cells (CD45^+^ CD3^+^ CD8^+^) **C.** show a significant increase in the erlotinib-treated (*n* = 3) *EGFR*^L858R^ mice compared to the vehicle-treated (*n* = 3) mice. Data are provided as mean +/− SEM. Statistical analysis: *p < 0.05 (Student's *t* test).

**Figure 10:**
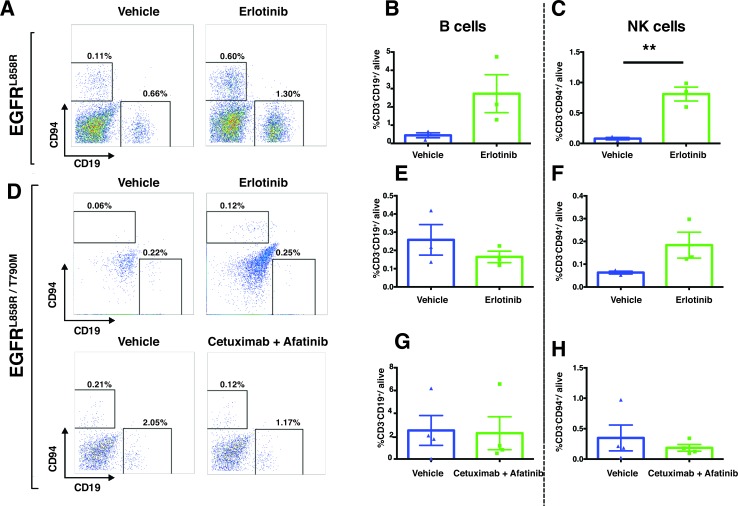
Increased NK cells in EGFR^L858R^ tumors treated with erlotinib Representative dot plots of NK cells (gated as CD45^+^ CD3^−^ CD94^+^) and of B cells (gated as CD45^+^ CD3^−^ CD19^+^) and their quantitation are shown in **A.**-**H. C.**, Flow-cytometry analysis of EGFR^L858R^- and EGFR^L858R/T790M^-driven tumors after erlotinib treatment (*n* = 3) shows increased NK cells in EGFR^L858R^ tumors compared to the vehicle-treated cohort (*n* = 3). Percentages of cells were shown relative to total live cells. Data are presented as mean +/− SEM. Statistical analysis: **p < 0.01 (Student's *t* test).

The immune compartment of the tumor microenvironment plays a major role in promoting tumor development. Since our findings described above suggest that EGFR-TKI therapy induces dramatic early responses in EGFR-addicted tumors, we sought to determine more precisely how EGFR-TKI impacts the lung-tumor immune microenvironment in the case of EGFR^L858R^-driven and EGFR^L858R/T790M^-driven tumors. In EGFR^L858R^-driven lung tumors, within 24 hours after treatment, CD45^+^ leukocytes increased significantly from 4% in vehicle-treated tumors to 18% in erlotinib-treated tumors (Figure 8A). However, there was no significant change of CD45^+^ leukocytes in EGFR^L858R/T790M^-induced tumors treated with either erlotinib or combination therapy (Figure 8B). In line with this, we also found that, among live cells in erlotinib-treated EGFR^L858R^-driven lung tumors, there was significantly increased infiltration of T lymphocytes (Figure 9A and Supplementary Figure 2), particularly of CD8^+^ T cells (Figure 9C) and of natural Killer (NK) cells (Figure 10A, and 10C). Notwithstanding, there was no significant change in these cell populations in the EGFR^L858R/T790M^-driven tumors treated either with erlotinib or with an afatinib-cetuximab combination (Figure 9B, 9D, 10D, 10F and 10H). However, there was no significant change in B cell population in both EGFR^L858R^-driven and EGFR^L858R/T790M^-driven tumors after the therapy (Figure 10A, 10B, 10D, 10E and 10G).

**Figure 11:**
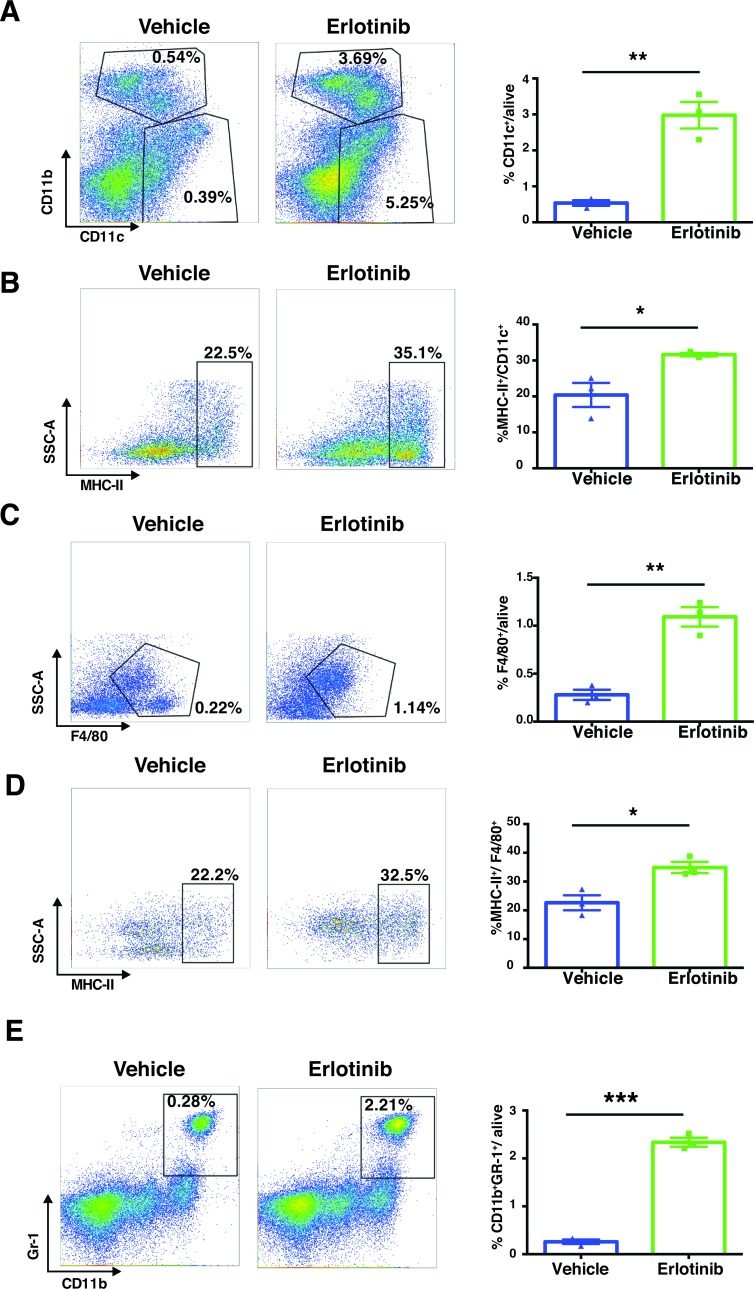
Erlotinib treatment of EGFR^**L858R**^ mice increased dendritic cells, macrophages and MDSCs within 24 hours **A.**, Dendritic cells (CD45^+^ CD11b^+^ CD11c^+^) were significantly increased in EGFR^L858R^-driven tumors that had been treated with erlotinib (*n* = 3) compared to those treated with vehicle (*n* = 3). **B.**, MHC-II expression within the DCs (MHC-II^+^/CD45^+^ CD11b^+^ CD11c^+^). **C.** and **D.**, The quantification of macrophages (CD45^+^ CD11b^+^ CD11c^−^ F4/80^+^) and MHC-II expression within macrophages (MHC-II^+^/CD45^+^ CD11b^+^ CD11c^−^ F4/80^+^) after vehicle (*n* = 3) or erlotinib treatment (*n* = 3). **E.**, Percentage of MDSCs (CD45^+^ CD11b^+^ Gr-1^+^) in EGFR^L858R^-driven tumors treated either with vehicle (*n* = 3) or with erlotinib (*n* = 3). Percentages of cells were shown relative to total live cells. Statistical analysis was performed by Student's *t* test and p values were denoted as following *p < 0.05, **p < 0.01 and data presented as mean +/− SEM.

Importantly, we found significant changes in the innate immune compartment of the lung tumor microenvironment in response to erlotinib treatment of EGFR^L858R^-driven tumors. We observed increased infiltration of dendritic cells (DCs), and macrophages, among live cells (Figure 11A and 11C). Upon erlotinib treatment, moreover, not only DCs (Figure 11B) but also macrophages (Figure 11D) expressed significantly higher MHC class II on their surfaces, showing increased antigen-presenting capabilities, in these APCs. We also saw an increased presence of myeloid-derived suppressor cells (MDSCs) in EGFR^L858R^-driven tumors treated with erlotinib (Figure 11E).

Altogether, erlotinib induces a striking infiltration of leukocytes within the tumor microenvironment of EGFR^L858R^-addicted lung tumors, an increase in T lymphocytes, dendritic cells, macrophages, NK cells, MDSCs and an enhanced MHC class II expression in dendritic cells and macrophages.

## DISCUSSION

In this study, we used EGFR TKI-sensitive and resistant mutant EGFR-driven transgenic mouse models to interrogate the immediate-early response to 1^st^- and 2^nd^-generation EGFR TKIs. Surprisingly, we demonstrated by MRI and FDG-PET imaging that EGFR^L858R^-driven tumors responded dramatically to erlotinib therapy within 24 hours after first treatment. In fact, radiographic tumor burden was reduced by about 65% within 24 hours of the initial dose. Tumor shrinkage as determined by MRI correlated with FDG-PET imaging response within 24 hours of erlotinib treatment in TKI-sensitive EGFR^L858R^ tumors. There was significant FDG-PET tumor response to afatinib-cetuximab combination treatment in EGFR^L858R/T790M^-driven tumors, but not to either erlotinib or afatinib monotherapy. In addition, we demonstrated that either erlotinib treatment of EGFR^L858R^-induced tumors or afatinib-cetuximab combination therapy applied to EGFR^L858R/T790M^ tumors resulted in significant induction of apoptosis and in reduced proliferation. In particular, Ttf^+^ tumor cells and EGFR^L858R^-expressing tumor cells were depleted in the early time period referred to above. Hence, we conclude that the observed MRI imaging response was due to dramatic regression of EGFR-addicted tumors in consequence of appropriate TKI treatment.

In addition, we investigated the early changes occurring in the tumor immune microenvironment within 24 hours consecutive to treatment, as above. We have thereby demonstrated a significant increase in CD45^+^ leukocytes upon erlotinib treatment in EGFR^L858R^-driven tumors within that time. We further analyzed these immune subsets and thus identified a significant increase in NK cells. So too, lymphocytes, particularly the CD8^+^ T cell population, were found to have increased in response to erlotinib in those tumors. Moreover, there was increased infiltration of dendritic cells and of macrophages. Importantly, these dendritic cells and macrophages had significantly higher MHC-II expression in response to erlotinib compared to vehicle treatment, suggesting increased antigen-presenting capabilities in these antigen-presenting cells (APCs). In summary, our study provides new insight both into the immediate-early therapeutic response to EGFR TKIs and into dynamic changes transpiring in the immune microenvironment during that time, phenomena which may mediate the tumor response.

Several clinical trials have demonstrated that NSCLC patients who present sensitizing *EGFR*-mutant tumors can respond dramatically to EGFR TKIs [8, 9, 18, 33, 34]. Moreover, pre-clinical studies have shown much that same type of response in mouse models when the latter harbor *EGFR*^L858R^ or *EGFR*^L858R/T790M^ transgenes and are treated with EGFR-targeted therapy [17, 20]. Nonetheless, immediate-early response to this overall category of treatment remains poorly understood and has not yet been investigated in adequate detail.

Our study suggests that tumors addicted to EGFR driver mutations respond in as soon as 24 hours when the driver mutation is targeted. As expected, however, erlotinib has proven ineffective against EGFR-TKI-resistant EGFR^L858R/T790M^-driven tumors [11, 35]. Previous studies, pre-clinical and clinical alike, have reported that tumors driven by EGFR^L858R/T790M^ exhibit: 1) only a modest response to monotherapy applied with the irreversible inhibitor afatinib; but 2) a significant response to afatinib-cetuximab combination therapy [15, 17, 18]. Likewise, in 24-hour imaging, we observed only a modest response to afatinib alone but a substantial one to combination therapy, which more effectively targets the EGFR^L858R/T790M^ mutant.

As measured by FDG-PET, and visualized by MRI, early tumor-imaging response has proven concordant with impeded EGFR downstream signaling and histopathological assessment. Therefore, the imaging responses predict early anti-tumor efficacy of EGFR-targeted treatment of EGFR-addicted tumors. Consistent with previous findings, moreover, we have demonstrated inhibition of EGFR downstream signaling. This finding was established by observing decreased phosphorylation of ERK, AKT and STAT3 phosphorylation alike in response to EGF-targeted therapy [17, 20, 36]. This suggests that the decreased activation of ERK, AKT and STAT3 may be inhibiting the pro-survival and anti-apoptotic signals, leading to an early response in the tumors themselves.

Multiple studies have suggested that the tumor microenvironment has a significant part in the mediation of treatment response [37–39]. It has been shown that erlotinib can potentiate lymphocyte migration through increased secretion of IL-2 as an “off-target” effect [40]. A majority of patients treated with erlotinib experience skin inflammation [41, 42]. Histopathological analysis of tumor-tissue sections has shown increase in immune infiltrates in erlotinib-treated lung tissues. This finding indicates that the tumor microenvironment does indeed have an important influence on treatment response. It has been proposed that increased apoptosis results in the release of damage-associated molecular patterns (DAMPs), increasing the antigen-presenting ability of dendritic cells and subsequently an effective T-cell response [43, 44]. In regard to this overall situation, it seems relevant that, in monitoring the response to erlotinib treatment in EGFR^L858R^ -induced lung tumors, we have also observed significantly increased MHC class II expression in APCs and an increased ratio of CD8^+^ T cells to total cells. Additionally, greater clearance of tumor cells in erlotinib-treated tumors could be associated with an increase in NK cells. Previous studies have shown that NK cells can remove MHC class I negative tumor cells rapidly, and erlotinib treatment increases NK-cell cytotoxicity [45, 46]. However, neither monotherapy nor afatinib-cetuximab combination resulted in any significant increase in immune infiltration in EGFR^L858R/T790M^ -induced lung tumors. This may be explained by the different histopathological characteristics of the lung tumors in these mouse models. EGFR^L858R/T790M^-induced tumors are nodular, surrounded by relatively normal alveoli, compared to the EGFR^L858R^-induced tumors that are diffuse with more broncho-alveolar pattern. We also speculate that acquired resistance to erlotinib by the emergence of T790M mutation in addition to L858R may result in a poorly immunogenic tumor as a result of differential immunoediting in the immunocompetent mouse models [47]. Therefore, although afatinib-cetuximab combination treatment induced tumor cell death in EGFR^L858R/T790M^ tumors resulting in reduced tumor burden, the effect was relatively modest compared to the effect of erlotinib treatment of EGFR^L858R^-induced tumors. We hypothesize that the antitumor effect of the combination treatment in the TKI-resistant tumors is predominantly a result of the combination anti-EGFR therapy than a combined effect with immune modulation.

We have identified an increased infiltration of MDSCs upon erlotinib treatment in EGFR^L858R^-driven tumors. Studies have shown that MDSCs orchestrate resistance to targeted therapy [38, 48]. Even though we have not functionally characterized these MDSCs, it has also been shown that, decreased STAT3 phosphorylation in MDSCs inhibits their suppressive activity [49]. Albeit we have not specifically investigated the STAT3 phosphorylation in MDSCs, we have analyzed pSTAT3 from whole tumors which included MDSCs. This analysis demonstrated that, upon erlotinib treatment, pSTAT3 decreased significantly. This signal attenuation is partially due to erlotinib inhibition of EGFR pathways in tumor cells. Further characterization of MDSCs is required in order to elucidate their role in tumor response.

In conclusion, we demonstrated dramatic response to therapy within 24 hours in response to erlotinib treatment in EGFR^L858R^-driven tumors and afatinib-cetuximab combination therapy response in EGFR^L858R/T790M^-driven tumors. Importantly, erlotinib induced a strong immune response as early as 24 hours post treatment. Immune infiltration was lower in EGFR^L858R/T790M^ -driven tumors. Our data provide rationale and a model for addressing early TKI responses in lung cancer, as well as raises the paradigm of how immediate-early tumor responses to EGFR-TKIs in “pre-clinical” *in vivo* models predicts long term therapy response in human lung adenocarcinoma.

## MATERIALS AND METHODS

### Mouse strains

Doxycycline-inducible transgenic *EGFR*^L858R^ and *EGFR*^L858R/T790M^ models were bred and propagated as done previously [4, 20]. All mice were maintained in pathogen-free facilities at the National Cancer Institute (NCI) and Memorial Sloan-Kettering Cancer Center (MSKCC) according to the approved protocols of the Animal Care and Use Committee (ACUC) of NCI and MSKCC.

### Ethics statement

Investigation has been conducted in accordance with the ethical standards and according to the Declaration of Helsinki and according to national and international guidelines and has been approved by the NCI and MSKCC review boards.

### Mouse-tumor monitoring

Tumors were generated in appropriate bi-transgenic mice, specifically in *CCSP^rtTA^/EGFR^L858R^ and in CC10^rtTA^/EGFR^L858R/T790M^,* by feeding them doxycycline-impregnated feed. Mice were monitored *via* MRI for tumor progression and treatment response. All experiments at the NIH Mouse Imaging Facility were performed on a 7T Bruker Biospec. Anesthesia was induced in a flow chamber with 5% isoflurane in medical air. Once the animal was fully anesthetized, it was transferred to a specially constructed cradle equipped with a face mask and anesthesia was maintained *via* the face mask. The gas was adjusted to maintain a breath rate of approximately 40 breaths per minute - typically 1-2% isoflurane. Breathing, heart rate, and temperature monitors were attached and the animal was placed in an MRI probe and then into the MRI scanner. Following acquisition of a set of locator images, a set of axial and coronal images were acquired covering the lungs using a FLASH sequence. The imaging parameters were TR/TE = 200/2.4ms, slice thickness = 1mm, field of view = 3cm, number of averages = 8, and imaging matrix = 256×256. Acquisition was gated to the ECG and breathing signals in order to reduce motion artifacts. Tumor burden was determined for the coronal slices by Region of Interest analysis using ImageJ as follows. For each slice, the lung and the tumor affected tissues were traced manually. The tumor burden was then calculated using the areas and intensities of the segmented regions.

### PET imaging

FDG-PET studies were performed using an Inveon small-animal PET scanner (Siemens Medical Solutions). ^18^F-FDG was administered intravenously to experimental mice with a dose of 3.7 MBq (100μCi) under isoflurane anesthesia. All the mice were imaged at 1 hour after injection for a 5 min static scan. The images were reconstructed using a two-dimensional ordered-subset expectation maximum (2D OSEM) algorithm, and no correction was applied for attenuation or scatter. For each scan, regions of interest (ROIs) were drawn using vendor software (ASI Pro 5.2.4.0) on decay-corrected whole-body coronal images. The radioactivity concentrations (accumulation) over the tumor regions were obtained from mean pixel values within the multiple ROI volume and then converted to MBq per milliliter. These values were then divided by the administered activity to obtain (assuming a tissue density of 1 g/ml) an image-ROI-derived percent-injected dose per gram (%ID/g). All images of the 0-hour treatments and 24-hour treatments were presented on the same scale. Relevant details on the mice used in PET imaging are shown in Supplementary Table 1.

### Histology and immunohistochemistry staining

The animals were sacrificed immediately after imaging at 24 hours of EGFR-TKI treatment. The lungs were perfused with cold PBS supplemented with phosphatase and kinase inhibitors. The left lung was flash-frozen in liquid nitrogen for immunoblot, and the rest of the lung tissue was inflated with 4% paraformaldehyde in PBS. The lungs were fixed in 4% paraformaldehyde overnight at room temperature and placed in 70% ethanol before paraffin embedding. Serial sections of the lungs were obtained. The sections were stained with hematoxylin and eosin and probed with TTF (Dako), EGFR^L858R^ (Cell signaling Technology), pERK (Cell signaling Technology) and Ki67 (Abcam). TUNEL staining was performed using the Apoptag Peroxidase *In situ* Apoptosis detection kit (Millipore).

### Flow cytometry

The mice were sacrificed and their lungs there upon perfused with cold PBS. Lungs of experimental mice were resected, and one left lobe was used for flow-cytometric analysis as described previously [50]. Tumor-bearing tissues were dissociated by means of a Tumor Dissociation Kit (Miltenyi Biotec) according to the manufacturer's instructions. Briefly, those lungs harboring diffuse or micro-dissected lung tumor nodules were shredded fine and incubated with the enzyme mix for forty minutes at 37°C with continuous rotation using the MACSmix Tube Rotator. Samples were collected after a short spin at 300g and then filtered using a 70-μm nylon mesh. The cells were re-suspended in FACS Buffer (PBS with 0.5%BSA) and subsequently stained with antibodies. Propidium iodide and LIVE/DEAD Violet (Invitrogen) were used as cell-death exclusion reagents. (Rat anti-mouse antibodies were purchased from BioLegend.) Flow cytometry was performed using a MACSQuant Analyzer equipped with MACSQuant VYB software (Miltenyi Biotec) and a BD Fortessa equipped with FACSDiva software (BD Biosciences). Data were analyzed *post hoc* using FlowJo (FlowJo, LLC.). The gating strategy used for flow cytometry is shown in Supplementary Table 2.

### Immunoblots

Murine lung adenocarcinoma tissue lysates were prepared and analyzed by immunobloting as previously described [51]. Briefly, using modified RIPA lysis buffer with protease and phosphatase inhibitors, we lysed the tissues using the tissue lyser (Qiagen) according to manufacturer's protocol. Total proteins were measured using a modified Lowry method. The lysates were denatured *via* the SDS loading buffer, and SDS-polyacrylamide (4-15%) gel electrophoresis was carried out. Proteins were transferred to nitrocellulose membrane and probed with antibodies against the proteins discussed in the figures. We developed the blots by chemiluminescence using Odessey Fc Imager (Licor). The following antibodies were obtained from Cell Signaling Technology: EGFR; pEGFR(Y1068); AKT; pAKT(T308); ERK; pERK; STAT3; and pSTAT3. Pro-SPC was procured from Millipore and ACTIN from Sigma-Aldrich.

### Statistical analysis

Student *t* tests were performed to assess the significance of before-and-after-therapy change as determined by MRI or PET imaging. For comparisons between two cohorts, statistical analyses were performed using a Student *t* test, and *P* < 0.05 was considered significant. GraphPad Prism 6 (GraphPad) was employed for statistical analysis. All numerical data are presented as mean ± SEM. Symbols indicating statistical significance are as follows (unless indicated otherwise): *, *P* < 0.05; **, *P* < 0.01; ***, *P* < 0.005.

## SUPPLEMENTARY MATERIALS


