# Supplementary material for: EGFR-targeted therapy results in dramatic early lung tumor regression accompanied by imaging response and immune infiltration in EGFR mutant transgenic mouse models
Source: Oncotarget. 2016 Aug 2;7(34):54137–56. doi: 10.18632/oncotarget.11021 (PMC5338915; doi:10.18632/oncotarget.11021)
Supplement: Supplementary file 1 [file oncotarget-07-54137-s001.pdf]

EGFR-targeted therapy results in dramatic early lung tumor regression accompanied by imaging response and immune infiltration in EGFR mutant transgenic mouse models

Supplementary Material

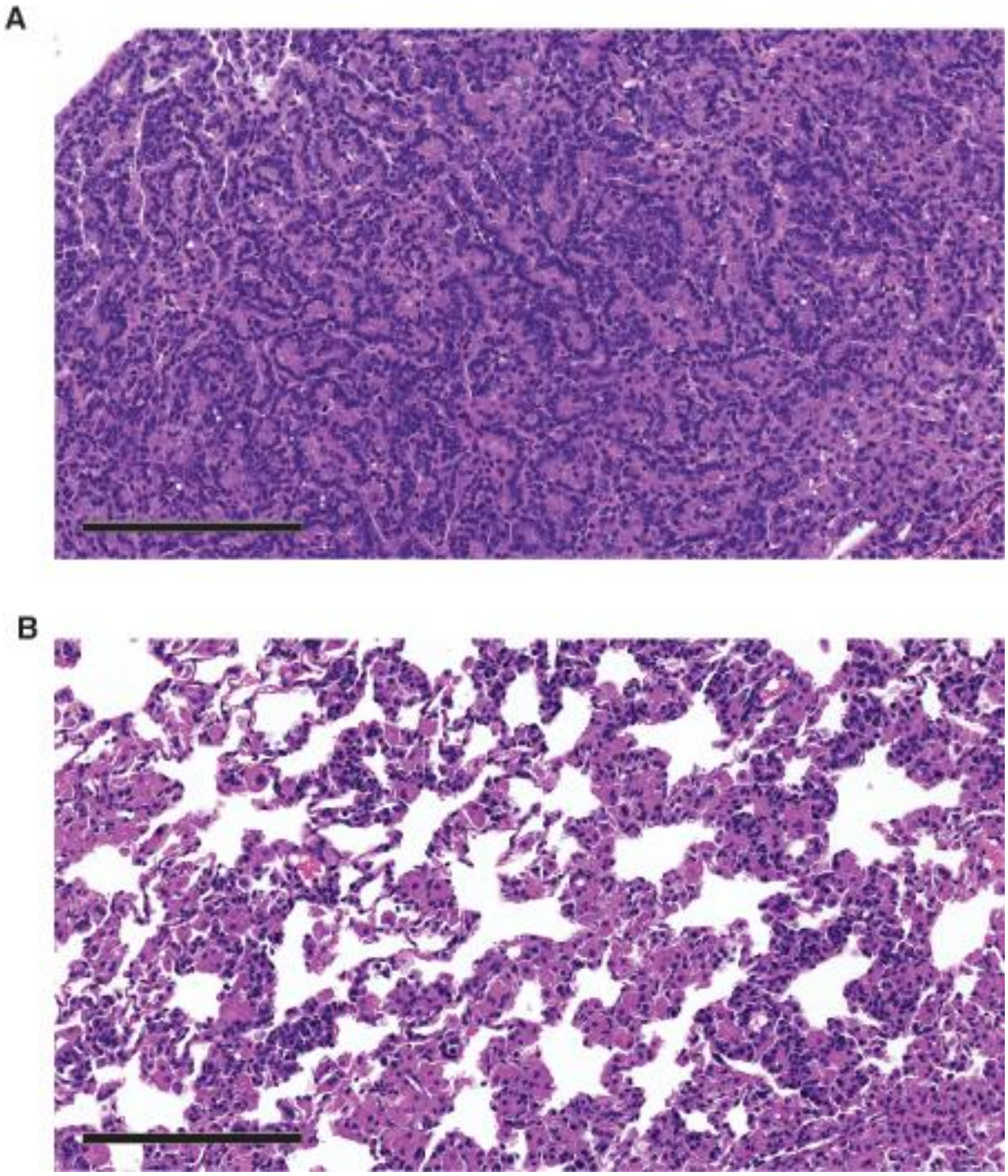

Supplementary Figure 1

Photomicrographs of $EGFR^{L858R}$-driven tumors treated with either vehicle (**A**) or erlotinib (**B**) show immune infiltration in erlotinib treated tumor sections. Scale bars, 200 μm.

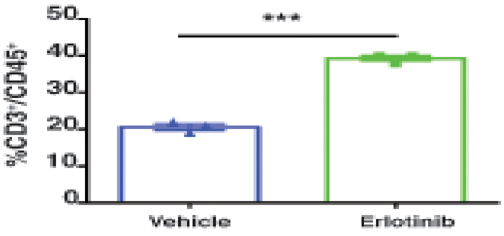

Supplementary Figure 2

CD3 positive immune subset as a % of CD45 positive cells. T lymphocytes ($CD3^+/CD45^+$) increased significantly after erlotinib treatment in $EGFR^{L858R}$-driven tumors. Statistical analysis: ***p < 0.001 (Student's *t* test) and data presented as mean +/- SEM.

Supplementary Table 1.<br><br>Details of mice used in immunoblot, FDG-PET and flow cytometry experiments					
**Designation**	**Mouse Number**	**Transgene**	**Drug Treatment**	**Days on Dox**	**Experiment**
M1	3238	$EGFR^{L858R}$	Vehicle	106	FDG-PET
M2	3271	$EGFR^{L858R}$	Vehicle	106	FDG-PET
M3	3264	$EGFR^{L858R}$	Vehicle	106	FDG-PET
M4	3207	$EGFR^{L858R}$	Vehicle	106	FDG-PET
M5	2801	$EGFR^{L858R}$	Erlotinib	155	FDG-PET
M6	2892	$EGFR^{L858R}$	Erlotinib	71	FDG-PET
M7	2893	$EGFR^{L858R}$	Erlotinib	155	FDG-PET
M8	2894	$EGFR^{L858R}$	Erlotinib	155	FDG-PET
M9	2840	$EGFR^{L858R}$	Erlotinib	155	FDG-PET
M10	3512	$EGFR^{L858R}$	Vehicle	134	Flow cytometry
M11	3406	$EGFR^{L858R}$	Vehicle	148	Flow cytometry
M12	3514	$EGFR^{L858R}$	Vehicle	134	Flow cytometry
M13	3537	$EGFR^{L858R}$	Vehicle	134	Flow cytometry
M14	3413	$EGFR^{L858R}$	Erlotinib	148	Flow cytometry
M15	3507	$EGFR^{L858R}$	Erlotinib	134	Flow cytometry
M16	3515	$EGFR^{L858R}$	Erlotinib	134	Flow cytometry
M17	3559	$EGFR^{L858R}$	Erlotinib	134	Flow cytometry
M18	3071	$EGFR^{L858R/T790M}$	Erlotinib	93	FDG-PET
M19	3072	$EGFR^{L858R/T790M}$	Erlotinib	93	FDG-PET
M20	3009	$EGFR^{L858R/T790M}$	Afatinib	96	FDG-PET
M21	3010	$EGFR^{L858R/T790M}$	Afatinib	93	FDG-PET
M22	3039	$EGFR^{L858R/T790M}$	Afatinib	93	FDG-PET
M23	3011	$EGFR^{L858R/T790M}$	Erlotinib	96	FDG-PET
M24	3038	$EGFR^{L858R/T790M}$	Afatinib	96	FDG-PET
M25	3070	$EGFR^{L858R/T790M}$	Afatinib	93	FDG-PET
M26	3146	$EGFR^{L858R/T790M}$	Cetuximab + Afatinib	152	FDG-PET
M27	3147	$EGFR^{L858R/T790M}$	Cetuximab + Afatinib	152	FDG-PET
M28	3221	$EGFR^{L858R/T790M}$	Cetuximab + Afatinib	152	FDG-PET
M29	3046	$EGFR^{L858R/T790M}$	Cetuximab + Afatinib	170	FDG-PET
M30	3103	$EGFR^{L858R/T790M}$	Vehicle	107	Flow cytometry
M31	3105	$EGFR^{L858R/T790M}$	Vehicle	107	Flow cytometry
M32	3144	$EGFR^{L858R/T790M}$	Vehicle	89	Flow cytometry
M33	3149	$EGFR^{L858R/T790M}$	Erlotinib	89	Flow cytometry
M34	3102	$EGFR^{L858R/T790M}$	Erlotinib	107	Flow

					cytometry
M35	3041	$EGFR^{L858R/T790M}$	Erlotinib	89	Flow cytometry
M36	3346	$EGFR^{L858R/T790M}$	Vehicle	103	Flow cytometry
M37	3347	$EGFR^{L858R/T790M}$	Vehicle	103	Flow cytometry
M38	3217	$EGFR^{L858R/T790M}$	Vehicle	113	Flow cytometry
M39	3046	$EGFR^{L858R/T790M}$	Vehicle	113	Flow cytometry
M40	3048	$EGFR^{L858R/T790M}$	Cetuximab+Afatinib	113	Flow cytometry
M41	3348	$EGFR^{L858R/T790M}$	Cetuximab+Afatinib	103	Flow cytometry
M42	3218	$EGFR^{L858R/T790M}$	Cetuximab+Afatinib	113	Flow cytometry
M43	3309	$EGFR^{L858R/T790M}$	Cetuximab+Afatinib	103	Flow cytometry

Supplementary Table 2

Gating strategy for identifying various immune cell types using flow cytometry

Immune cells	Gating markers
T cells	$CD45^+ CD3^+$
B cells	$CD45^+ CD3^- CD19^+$
NK cells	$CD45^+ CD3^- CD94^+$
Macrophages	$CD45^+ CD11b^+ CD11c^- F4/80^+$
Myeloid-derived suppressor cells (MDSCs)	$CD45^+ CD11b^+ Gr\text{-}1^+$
Dendritic cells	$CD45^+ CD11b^+ CD11c^+$
